# Endothelial Piezo1 stimulates angiogenesis to offer protection against intestinal ischemia–reperfusion injury in mice

**DOI:** 10.1186/s10020-025-01197-3

**Published:** 2025-04-22

**Authors:** Cuifen Wang, Shangfei Luo, Yameng Yan, Jinze Li, Weipin Niu, Tianying Hong, Kai Hao, Xin Sun, Jiali Liu, Ran An, Jing Li

**Affiliations:** 1 Innovation Research Center, Shandong University of Chinese Medicine, Jinan, 250307 China; 2 The First Affiliated Hospital, Guangzhou University of Chinese Medicine, Guangzhou, 510405 China; 3 Pacific College of Health and Science, 110 William St 19 th Floor, New York, NY 10038 USA

**Keywords:** Intestinal I/R injury, Piezo1, Endothelial cells, Angiogenesis, HIF-1α

## Abstract

**Background:**

Intestinal ischemia–reperfusion (I/R) injury, which occurs in the ileum and not only leads to intestinal tissue damage, but also may trigger systemic inflammatory responses, is a prevalent pathological condition that is typically associated with acute intestinal ischemia, surgical procedures, or trauma. However, the precise underlying pathogenic mechanisms have not yet been fully uncovered. In this study, we explored the specific roles and underlying mechanisms by which endothelial Piezo1 is involved in intestinal I/R injury.

**Methods:**

We evaluated the roles of Piezo1 using both in vivo mouse intestinal ischemia–reperfusion (I/R) injury and in vitro hypoxia-reoxygenation (H/R) models. The expression of Piezo1 was assessed using immunofluorescence and RT-qPCR. In vivo and in vitro experiments involving endothelial knockout and activation of Piezo1 with the specific agonist Yoda1 were conducted to observe the effects on angiogenesis and injury.

**Results:**

We found that in post-intestinal I/R mice, Piezo1 expression was markedly increased and was mainly abundant in ileum endothelial cells. Specific knockout of endothelial *Piezo1* exhibited a more severe phenotype characterized by accelerating damage to the ileum structure, increasing inflammatory response, and inhibiting angiogenesis. Yoda1-mediated activation of Piezo1 significantly ameliorated intestinal I/R injury. Activation of Piezo1 induced by Yoda1 or H/R promoted angiogenesis in Human Umbilical Vein Endothelial Cells (HUVECs), which was inhibited by GsMTx4. Piezo1 mediated endothelial angiogenesis was linked to an increase of extracellular Ca^2+^ influx, which in turn enhanced hypoxia-inducible factor 1 alpha (HIF-1α) signaling pathway.

**Conclusions:**

Our findings indicate that Piezo1 plays a crucial role in protecting against intestinal I/R injury by promoting angiogenesis in endothelial cells, possibly through the activation of the Ca^2+^/HIF-1α/VEGF signaling pathway. This suggests that targeting endothelial Piezo1 channels could be a therapeutic strategy for ileum I/R injury.

**Supplementary Information:**

The online version contains supplementary material available at 10.1186/s10020-025-01197-3.

## Introduction

Intestinal I/R injury serves as a severe pathological process, frequently contributing to significant morbidity and mortality, particularly in conditions such as acute mesenteric ischemia, major surgeries involving abdominal vasculature, and inflammatory bowel disease (Li et al. 2021). The defining characteristic of I/R injury is the disruption of intestinal microcirculation, resulting in oxygen and nutrient deprivation. Subsequently, the reintroduction of oxygen initiates a cascade of inflammatory and oxidative stress responses, which leads to septicemia, potentially progressing to multi-organ failure (Watson et al. 2008; Deng et al. 2022). During I/R injury, the intestinal microvascular endothelium, functioning as a dynamic and responsive interface and embodying a complex and multifaceted physiological process, underlies a significant portion of the pathology associated with this condition (Bentley and Chakravartula 2017). The endothelium, being in direct contact with the circulating blood, is among the first cellular constituents to sense and respond to the changes in oxygenation and nutrient delivery during I/R damage.

Endothelium holds a central position in angiogenesis, which is indispensable for the restoration and healing of ischemic tissue (Chiba et al. 2021). The development of new blood vessels is tightly controlled by the release of angiogenic factors, such as vascular endothelial growth factor (VEGF), fibroblast growth factor (FGF) and intercellular cell adhesion molecule-1 (ICAM-1), which are activated in response to hypoxic conditions (Mendola et al. 2022). Endothelium then undergoes a series of complex changes, including proliferation, migration, and tube formation, to establish a new vascular network that can restore blood flow to the injured tissues (Wei et al. 2023).

The control of vascular growth, known as angiogenesis, is a sophisticated process that entails maintaining a fine balance between factors that promote and those that inhibit vessel formation (Dudley and Griffioen 2023). Mechano-transduction, the intricate process whereby mechanical forces are transformed into biochemical signals, is important in regulating vascular homeostasis and angiogenesis. Mechanical forces, such as shear stress and tissue stiffness, are recognized to exert an influence on endothelial cell function and vascular remodeling processes (Kang et al. 2019). Piezo1, as a non-selective Ca^2+^ channel embedded in cell membrane, is a key sensor of mechanical forces and has been implicated in processes of physiology and pathology, including vascular development, iron metabolism, atherosclerosis and tissue fibrosis (Lai et al. 2022; Luo et al. 2023; Ma et al. 2021). Abnormal mechanical forces stimulating the endothelium lead to the activation of Piezo1, resulting in an influx of Ca^2+^ that initiates a cascade of cellular responses, encompassing the secretion of angiogenic factors and the engagement of signaling pathways vital for cell migration and proliferation (Wang et al. 2019). Recent research has indicated that Piezo1 occupies a pivotal position in angiogenic response associated with irradiation injury and embryonic development (Zhang et al. 2022; Li et al. 2014).

Abnormal blood flow and other mechanical forces may activate Piezo1 during intestinal I/R injury, which subsequently promotes the formation of new blood vessels and aids in the restoration of tissue perfusion and function. The aim of our research was to explore the roles of endothelial Piezo1 in angiogenesis and its possible protective benefits in mitigating intestinal I/R injury. Piezo1 activation by mechanical forces during the reperfusion phase may trigger the secretion of angiogenic factors, ultimately resulting in new vascular formation and contributing to the alleviation of tissue damage caused by I/R injury. By employing I/R injury models in vitro and in vivo, we explored the mechanisms by which Piezo1 modulates angiogenic responses and evaluated the therapeutic benefits of targeting Piezo1 signaling in the setting of intestinal I/R injury. Our research findings could offer fresh perspectives on the mechanosensitive modulation of angiogenesis and suggest a novel approach for the prevention and management of intestinal I/R damage.

## Methods

### Animals

All animal experiments were approved by the Shandong University of Traditional Chinese Medicine Animal Ethics Committee (Approval number: STUDCM 20230428001). 8–10 weeks old male C57BL/6 J mice were purchased from Animal Laboratory Center of Shandong University of Traditional Chinese Medicine. Endothelial cell (EC) specific *Piezo1* knockout mice (*Piezo1*^*∆EC*^) were generated through the crossing of Cdh5 (PAC)-CreERT2 transgenic mice (University of Münster, Germany) and *Piezo1*^*fl/fl*^ mice (No. 029213, from the Jackson Laboratories). The *tdTomato* (*Piezo1*^*td/Tdt*^, No. 026948, from the Jackson Laboratories) were used in this study. All mice were in C57BL/6 J background and were housed in a controlled environment within an SPF-level Laboratory Animal Room. The identification of *Piezo1*^*∆EC*^ mice was conducted through polymerase chain reaction (PCR) genotyping. The sequences of the PCR primers employed were as follows: mouse- *Cdh5* (Forward) 5ʹ-TGCCACCAGCCAGCTATCAACT-3ʹ, (Reverse) 5ʹ-AGCCACCAGCTTGCATGATCTC-3ʹ; mouse- *Loxp* (Forward) 5ʹ-GCCTAGATT CACCTGGCTTC-3ʹ, (Reverse) 5ʹ-GCTCTTAACCATTGAGCCATCT-3ʹ.

### Mice model and treatment

To induce knockout of *Piezo1*, male *Piezo1*^*∆EC*^ mice and their *Piezo1*^*fl/fl*^ littermates, approximately 6 weeks of age, were intraperitoneally administered tamoxifen (Sigma) at a dosage of 75 mg/kg for five consecutive days. Further experiments were conducted one week following the final tamoxifen injection.

The murine model of intestinal I/R injury was established according to a previously published methodology (Gubernatorova et al. 2016). In brief, male mice aged 10 and 12 weeks were fasted for 12 h before surgery, with water provided ad libitum. Following surgical site preparation, the mice were rendered unconscious through inhalation of 2% isoflurane. A 1–2 cm incision was made along the abdominal midline, then abdominal layers were sequentially separated. The first-order branch of the superior mesenteric artery was isolated using blunt dissection techniques. Mesenteric ischemia was induced by clamping the ileal vessels with a microvascular clamp, and successful induction was verified by observing the resultant pallor of the ileum. After 30 min of ischemia, the clamp was taken away, and the abdominal wound was sutured. The mice were euthanized at 48 and 72 h post-reperfusion for further analysis.

For Yoda1 (MCE, Shanghai, China) treatment, a 50 mM stock solution was prepared by dissolving Yoda1 in dimethyl sulfoxide (DMSO). For experimental use, the saline was used to dilute the stock solution to achieve a final concentration of 5 µmol/kg. Mice were continuously injected with Yoda1 intraperitoneally for 5 days one week before surgery.

### Pathological analysis

Tissue slices (4 μm) of 4% paraformaldehyde-fixed, paraffin-embedded ileal tissues were deparaffinized, hydrated and then stained with Hematoxylin & eosin (H&E) and Alcian Blue-Periodic Acid-Schiff (AB-PAS) commercial kits according to the manufacturer’s instructions. Assessment of intestinal mucosal damage after I/R using Chiu Score based on H&E staining.

### Cell culture

HUVECs were seeded into 6-well plates and cultured in DMEM medium containing 10% Fetal Bovine Serum (FBS), 4 μg/mL VEGF, and 1% Penicillin/Streptomycin (P/S). The cells were then incubated at 37 ℃ in an atmosphere containing 5% CO_2_. After reaching 80% confluence, HUVECs were stimulated with 5 μM Yoda1 or 10 μM GsMTx4 (MCE, Shanghai, China) for 12 h.

To mimic I/R damage, HUVECs were treated with H/R conditions: they were cultured under anaerobic conditions for 48 and 72 h, followed by reoxygenation for 2 h.

### Isolation of primary murine intestinal endothelial cells

To isolate primary intestinal endothelial cells, small intestinal tissues were extracted from 8-week-old *Piezo1*^*∆EC*^ and *Piezo1*^*fl/fl*^ mice and carefully divided into segments, approximately 0.5–1 cm in length. The minced intestinal segments were then transferred into pre-chilled Dulbecco's phosphate-buffered saline (D-PBS), enriched with antibiotics, to effectively eliminate luminal contents and external debris. Afterward, the intestinal segments were transferred to a container filled with a digestion mixture of 0.1% collagenase I and 0.3% Dispase II, and incubated at 37 °C for 25 min. After finishing digestion, a Vortex oscillator was used to thoroughly resuspend the digested tissue. The resultant mixture was filtered through a 100 µm mesh filter to remove any remaining undigested tissue fragments, enabling the collection of cells from the filtrate. The cells were collected into a pellet by centrifuging the cell suspension at 500 g for 10 min at 4 °C. The cell pellet was washed three times with PBS, resuspended in ECM medium, and plated onto culture dishes. The dishes were then incubated at 37 °C with 5% CO_2_.

### *Intracellular Ca*^*2*+^*measurements*

The culture of primary intestinal endothelial cells was conducted using transparent 96-well plates. Subsequently, the SBS solution was prepared, containing 1.5 mM CaCl_2_, 8 mM glucose, 1.2 mM MgCl_2_, 5 mM KCl, 130 mM NaCl, and 10 mM HEPES, and the pH was adjusted to 7.4 using NaOH. Cells were incubated with SBS containing 2 µM Fura-2 AM (ThermoFisher Scientific, USA) and 0.1% Pluronic acid at 37 °C in the dark for 1 h. After the incubation period, the cells were gently rinsed twice with SBS. The intracellular Ca^2+^ level alterations were measured using a FlexStation3 microplate reader following administration of 5 μM Yoda1, with quantitative determination achieved through calculating the ratio of emission intensities recorded at 340 and 380 nm excitation wavelengths using Fura-2 calcium indicator.

HUVECs were co-incubated with Fluo-4 AM (4 μM; Solarbio, China) for 20 min at 37 ℃ according to the reagent instructions. Afterward, the cells underwent two washes with Hanks buffer and were subsequently resuspended in Hanks buffer containing 1% FBS at 37 °C for 40 min. Imaging was performed using a fluorescence microscope.

### Real-time quantitative PCR (RT-qPCR)

Total RNA from mice ileum tissues, HUVECs and primary intestinal endothelial cells were isolated using Trizol (Sparkjade, Shandong, China) followed by the manufacturer’s protocol. After synthesizing cDNA using a commercial kit (Sparkjade, Shandong, China), RT-qPCR was conducted utilizing a LochightCycler 480II system (Switzerland, Germany), employing the reactions provided by the qRT-PCR Kit (Sparkjade, Shandong, China). The sequences of the PCR primers were showed in Table 1.

**Table 1:** Primer sequences

Gene	Species	Forward sequence	Reverse sequence
*Vegfa*	Mouse	GCACATAGAGAGAATGAGCTTCC	CTCCGCTCTGAACAAGGCT
*Il6*	Mouse	CAACGATGATGCACTTGCAGA	TGTGACTCCAGCTTATCTCTTGG
*Il1b*	Mouse	AGCTCTCCACCTCAATGGAC	GACAGGCTTGTGCTCTGCTT
*Tnf*	Mouse	GCACTCCCCCAAAAGATG	TGGTGGTTTGTGAGTGTGAG
*Piezo1*	Mouse	CTTACACGGTTGCTGGTTGG	CACTTGATGAGGGCGGAAT
*Fgf2*	Mouse	AACGGCGGCTTCTTCCTG	TGGCACACACTCCCTTGATAG
*Icam1*	Mouse	GTGATGCTCAGGTATCCATCCA	CACAGTTCTCAAAGCACAGCG
*Hif1a*	Mouse	CGCCTCTGGACTTGTCTCTT	TCGACGTTCAGAACTCATCC
*Actb*	Mouse	CAGCCTTCCTTCTTGGGTATG	AGCTCAGTAACAGTCCGCCT
*HIF1 A*	Human	CAGAAGATACAAGTAGCCTC	CTGCTGGAATACTGTAACTG
*VEGFA*	Human	CGAAAGCGCAAGAAATCCCG	GCTCCAGGGCATTAGACAGC
*ACTB*	Human	GATTCCTATGTGGGCGACGA	AGGTCTCAAACATGATCTGGGT

The relative expression levels were calculated using the 2^−ΔΔct^ method.

### Enzyme-linked immunosorbent assay (ELISA)

From each mouse, collect ileum tissue and add PBS in a mass ratio of 1 part tissue to 10 parts PBS (1:10). Utilizing a grinder to homogenize the tissue until a homogeneous mixture is obtained, centrifuging the homogenate at 3000 rpm at 4 °C for 15 min, and then collecting the supernatant. The levels of TNF-α, IL-6, and IL-1β in ileal tissues were quantified using ELISA Kits from Biolegend (CA, USA). The OD value was obtained at a wavelength of 450 and 570 nm.

### Wound healing assay

HUVECs were plated into 6-well dishes and cultured until reached 90% confluence. For the treatment groups, HUVECs were stimulated with either 5 μM Yoda1 or 10 μM GsMTx4 for 12 h. Subsequently, a standardized scratch was made across the surface of the plate using a 200-µL pipette tip, ensuring consistent pressure was applied throughout the process. The cells were gently rinsed three times with PBS and then cultured in DMEM medium enriched with 10% FBS, 4 μg/mL VEGF, and 1% P/S. The model group and the model plus drug group underwent H/R treatment, while the remaining groups were maintained under normal culture conditions. Photographs of the scratch wound sites were taken 48 h after the initial scratch.

### Tube formation assay

Cells were cultivated in 6-well plates under the aforementioned conditions. When confluence reached 80–90%, pharmacological intervention was initiated. After 12 h, the model group and the model plus drug group underwent H/R treatment, while the other groups were maintained under normal culture conditions. Subsequently, cells from all groups were reseeded into 96-well plates coated with a matrix adhesive agent. Following a 24 h incubation period, images of tubular structures in each well were captured using phase-contrast microscope.

### Immunofluorescence (IF) staining

After fixing with 4% paraformaldehyde, cells were thoroughly rinsed three times with PBS. To minimize any unwanted binding, a blocking procedure using 2.5% goat serum was implemented. Following this, the cells were incubated with the primary antibodies, including anti-VEGFR2 (1:100; Proteintech, 26415-1-AP, China), anti-HIF-1α (1:100; Abcam, ab179483, UK), anti-VEGF (1:100; Bioss, BS1313R, China), anti-Piezo1 (1:100; Proteintech, 15939-1-AP, China) overnight at 4 °C. Afterward, the cells were incubated with FITC conjugated Goat Anti-Rabbit IgG (1:200; Servicebio, GB22303) for 1 h at room temperature in the dark. The cells were then thoroughly washed three times with PBS, and the nuclei were counterstained with DAPI before being mounted with a sealing medium (Sparkjade, Shandong, China). Finally, the slides were captured using a fluorescence microscope (Zeiss, Germany).

For ileum samples, frozen slices were rewarmed, antigen retrieval was carried out at room temperature for 10 min, and subsequently, the slices were washed twice with PBS. The slices were then blocked with 5% goat serum for 1 h and incubated with anti-CD31 (1:100; Abcam, ab281583, UK), anti-RFP (1:200; Rockland) overnight at 4 °C, the primary antibodies were aspirated in the next day, and the slices underwent two washes with PBS. The slices were then incubated in the dark for 1 h with either FITC-conjugated Goat Anti-Rabbit IgG (1:200; Servicebio, GB22303), Cy3-conjugated Goat Anti-Rabbit IgG (1:200; Servicebio, GB22301), or Cy3-conjugated Goat Anti-Mouse IgG (1:200; Servicebio, GB21301). Subsequently, the slices were counterstained with DAPI for 5 min and washed with PBS.Finally, the slices were photographed using a high-end inverted fluorescence microscope.

### Western blot

HUVECs were treated with the respective drugs once the confluence reached 80%. After the treatment, the cells were washed twice with Phosphate-Buffered Saline (PBS) and then lysed on ice using Radio-Immunoprecipitation Assay (RIPA) lysis buffer containing Phenylmethylsulfonyl fluoride (PMSF) at a dilution of 1:100 and Protease Inhibitor Cocktail (PA) at a dilution of 1:1000 to release proteins. The cells were collected using a cell scraper and transferred to a 1.5 mL centrifuge tube. The samples were centrifuged at 4 °C at 12,000 rpm for 15 min. The supernatant was mixed with loading buffer at a ratio of 4:1 and heated at 100 °C for 15 min to denature the proteins, preparing them for subsequent experiments.

Protein samples were subjected to electrophoresis in sodium dodecyl sulfate polyacrylamide gels (SDS-PAGE) and then transferred to a polyvinylidene fluoride (PVDF) membrane (0.45 μm). The PVDF membrane was blocked with 5% skim milk. The primary antibodies, including anti-VEGF (1:1000, Bioss Biotechnology China, BS1313R), anti-HIF-1α (1:1000, Abcam, Cambridge, UK, ab179483), anti-Piezo1 (1:1000, Proteintech, 15939-1-AP, China) and anti-Tubulin (1:1000, Beyotime, China, AF1216) were incubated at 4 °C for 14 h. After washing with TBST, HRP-conjugated anti-rabbit IgG (1:5000, CST, 7074S) was incubated at room temperature for 1 h. Following three washes with TBST, the membrane was developed with an ECL solution, and the immunoblots were visualized using a chemiluminescence imaging system. The bands were quantified using ImageJ software.

### Statistical analyses

The data are presented as the mean ± SEM, where 'n' denotes the number of individual experimental trials. Unpaired two-tailed Student's *t*-tests were employed for comparisons between two groups, whereas one-way ANOVA, followed by Tukey's post-hoc test, was used for multiple group comparisons. The data presented in Figs. 2 and 3 were statistically analyzed using two-way ANOVA to evaluate significance under experimental conditions. No animal or sample data was excluded from the statistical analysis. Assignment of animals to treatment and control groups was randomized. Statistical significance was established at *P* < 0.05, indicated by an asterisk. Statistical analyses using OriginPro 2021 software.

## Results

### Expression of Piezo1 in ileal endothelium is upregulated post intestinal I/R injury

**Fig. 1:**
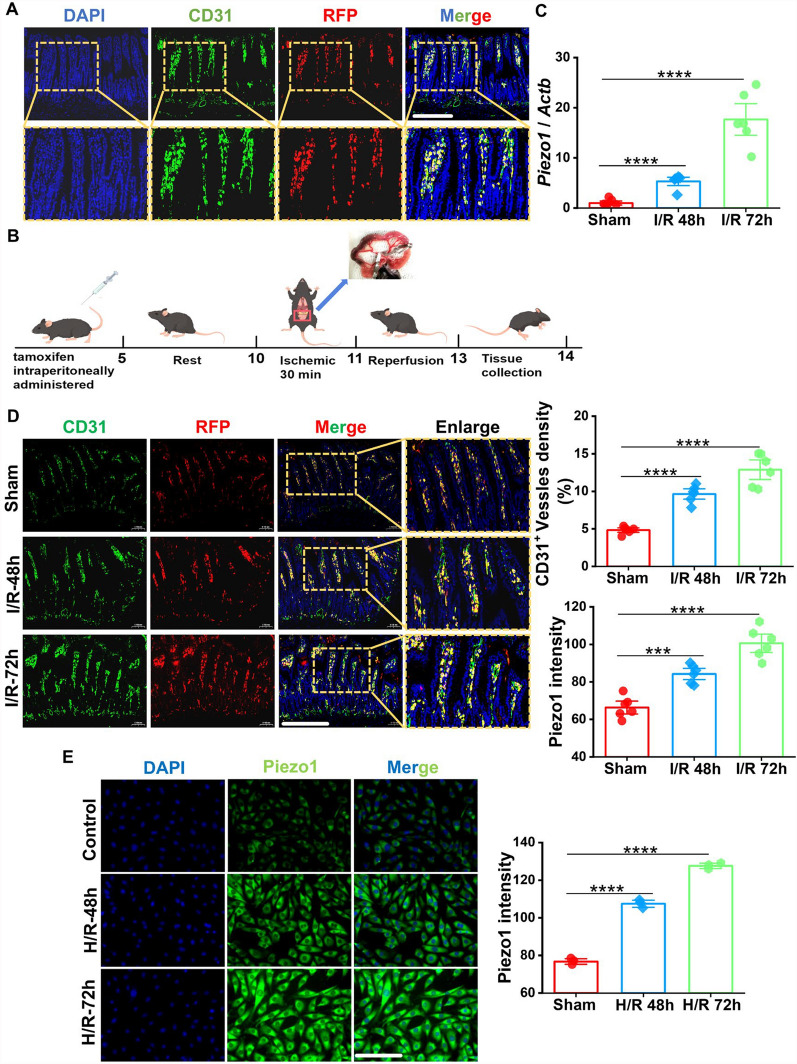
The expression of Piezo1 is upregulated post intestinal I/R injury. **a** Dual IF staining for CD31 (green) and Piezo1 (RFP, red) in ileal samples from *Piezo1*^*td/Tdt*^ mice. Scale bar, 100 µm; enlarge, 50 µm. **b** Schematic illustration of the intestinal I/R injury establishment in *Piezo1*^*td/Tdt*^ mice. **c** Relative mRNA expression of *Piezo1* in ileal tissues of *Piezo1*^*td/Tdt*^ mice (n = 6). **d** Representative images (left) of dual IF staining for CD31 (green) and Piezo1 (RFP, red) and quantification (right) of Piezo1 intensity and CD31 positive area in ileal tissues of *Piezo1*^*td/Tdt*^ mice (n = 6). Scale bar, 100 µm; enlarge, 50 µm. **e** Immunofluorescence detection (left) of Piezo1 (green) and quantification (right) of Piezo1 intensity in HUVECs after hypoxia for 48 and 72 h, followed by reoxygenation for 2 h (n = 3). Scale bar, 100 µm. Data are presented as mean ± SEM; ***p < 0.001, ****p < 0.0001, compared with Sham group

To investigate expression of Piezo1 following intestinal I/R damage, *Piezo1*^*td/Tdt*^ mice were employed to observe the expression level of Piezo1 channel. IF staining showed that Piezo1 was mainly expressed in CD31-positive cells, which are regarded as a marker of endothelial cells (Fig. 1a). To ascertain the dynamic expression of Piezo1 in intestinal post-I/R injury, ligation of the first-order branch of the superior mesenteric artery was performed in *Piezo1*^*td/Tdt*^ mice (Fig. 1b). RT-qPCR and IF analyses demonstrated upregulated levels of Piezo1 in the ileal tissues at both 48 and 72 h post-I/R (Fig. 1c, d). To validate this phenomenon in vitro, HEVECs were cultured under H/R conditions to mimic intestinal I/R injury. Similarly, IF results also substantiated that Piezo1 expression was notably higher in both 48 and 72 h–H/R groups than control group (Fig. 1e). Collectively, these findings indicated a pronounced elevation in Piezo1 expression in the ileum following I/R injury, suggesting a vital regulatory role for Piezo1 in the pathological processes of intestinal I/R injury.

### Knockout of endothelial Piezo1 exacerbates intestinal I/R injury

**Fig. 2:**
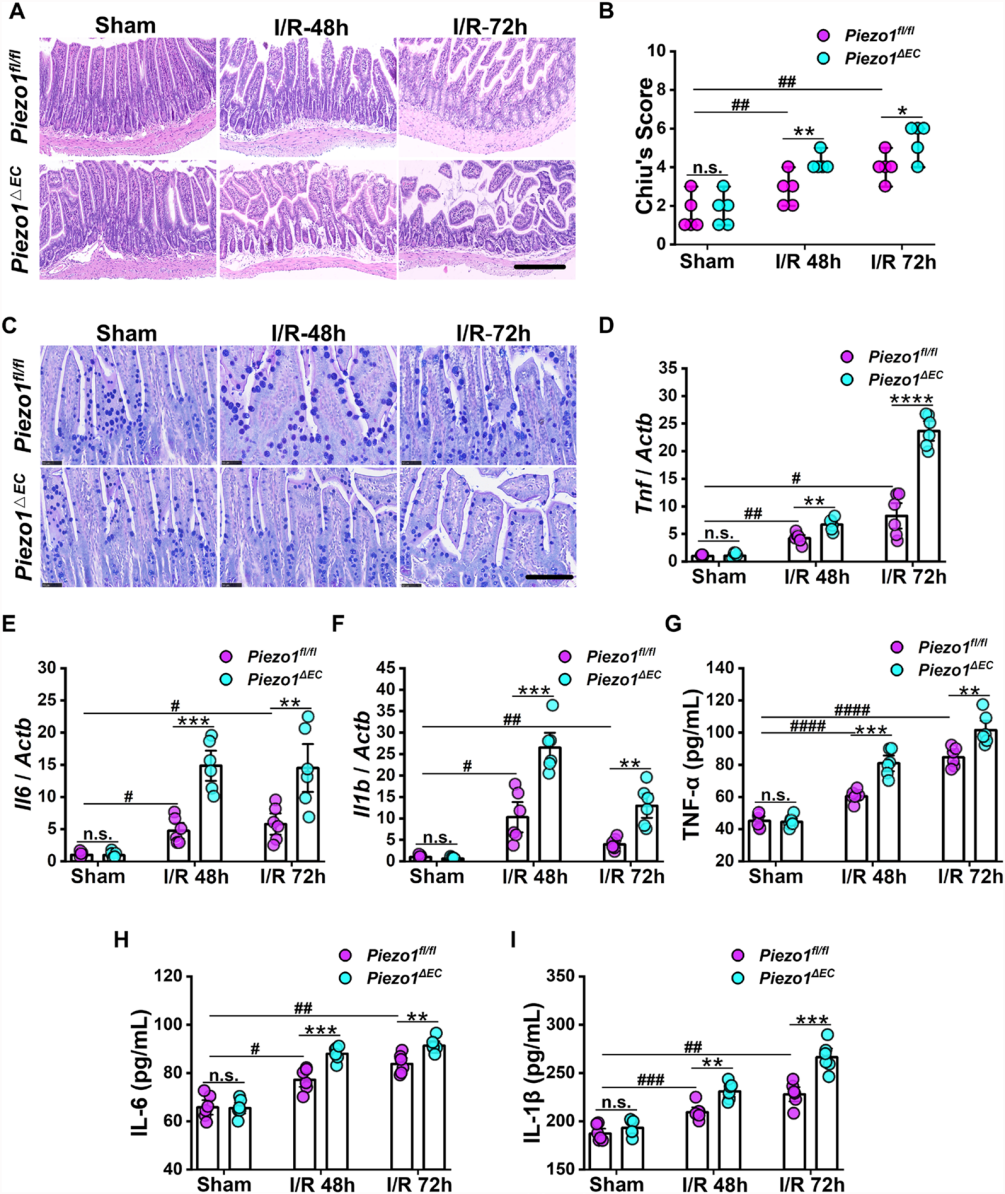
Knockout of endothelial *Piezo1* exacerbates intestinal I/R injury. **a** Representative images of H&E staining in ileal slices. Scale bar, 100 µm. **b** Histopathological scoring (Chiu's score) of ileal tissues as shown in (**a**) (n = 6). **c** Representative images of AB-PAS staining of ileal slices. Scale bar, 50 µm. **d–f** mRNA expression of *Tnf*, *Il6*, and *Il1b* in ileal tissues (n = 6). **g–i** The levels of TNF-α, IL-6, and IL-1β detected by ELISA in ileal tissues (n = 6). Data are presented as mean ± SEM. *p < 0.05, **p < 0.01, ***p < 0.001, ****p < 0.0001, compared with *Piezo1*^*fl/fl*^ group at the same time point; ^#^*p* < 0.05, ^##^*p* < 0.01, ^###^*p* < 0.001, ^####^*p* < 0.0001, compared with Sham *Piezo1*^*fl/fl*^ group

To investigate the roles of endothelial Piezo1 in intestinal post-I/R injury, endothelium-specific knockout of *Piezo1* (*Piezo1*^*∆EC*^) mice were utilized in this study. Genotyping results obtained through PCR analysis confirmed the presence of Cre enzyme in *Piezo1*^*∆EC*^ mice (Additional file 1: Fig. S1 A). After completing the intraperitoneal injection of tamoxifen, endothelial cells were extracted from intestinal tissues of *Piezo1*^*∆EC*^ mice and their littermates (*Piezo1*^*fl/fl*^) to verify the successful knockout of *Piezo1* in *Piezo1*^*∆EC*^ mice (Additional file 1: Fig. S1B–E). To determine whether the absence of *Piezo1* affects the process of intestinal I/R injury, experiments were conducted in *Piezo1*^*fl/fl*^ and *Piezo1*^*∆EC*^ mice. H&E staining revealed that the intestinal mucosa of sham-operated *Piezo1*^*fl/fl*^ mice was intact, with villi standing upright and neatly arranged, while villi were broken in 48 and 72 h post-I/R *Piezo1*^*fl/fl*^ groups. Moreover, compared to *Piezo1*^*fl/fl*^ mice, post-I/R *Piezo1*^*∆EC*^ mice exhibited exacerbated intestinal villus injury, with large areas of the villus structure missing, loss of basic architecture, and increased inflammatory cell infiltration (Fig. 2a). The Chiu's score in the ileal sections of post-I/R *Piezo1*^*∆EC*^ group was significantly higher compared to *Piezo1*^*fl/fl*^ group (Fig. 2b). In addition, the AB-PAS staining results confirmed a marked reduction in content of mucin in the ileum of post-I/R *Piezo1*^*∆EC*^ mice compared with *Piezo1*^*fl/fl*^ mice (Fig. 2c). Furthermore, post-I/R *Piezo1*^*∆EC*^ mice exhibited markedly elevated mRNA levels of *Tnf*, *Il6* and* Il1b* and protein levels of inflammatory factors in their ileal tissues, compared to *Piezo1*^*fl/fl*^ mice (Fig. 2d–i). These data suggested that knockout of endothelial *Piezo1* significantly worsens intestinal injury following I/R injury.

### Knockout of endothelial Piezo1 attenuates angiogenesis following intestinal I/R injury

**Fig. 3:**
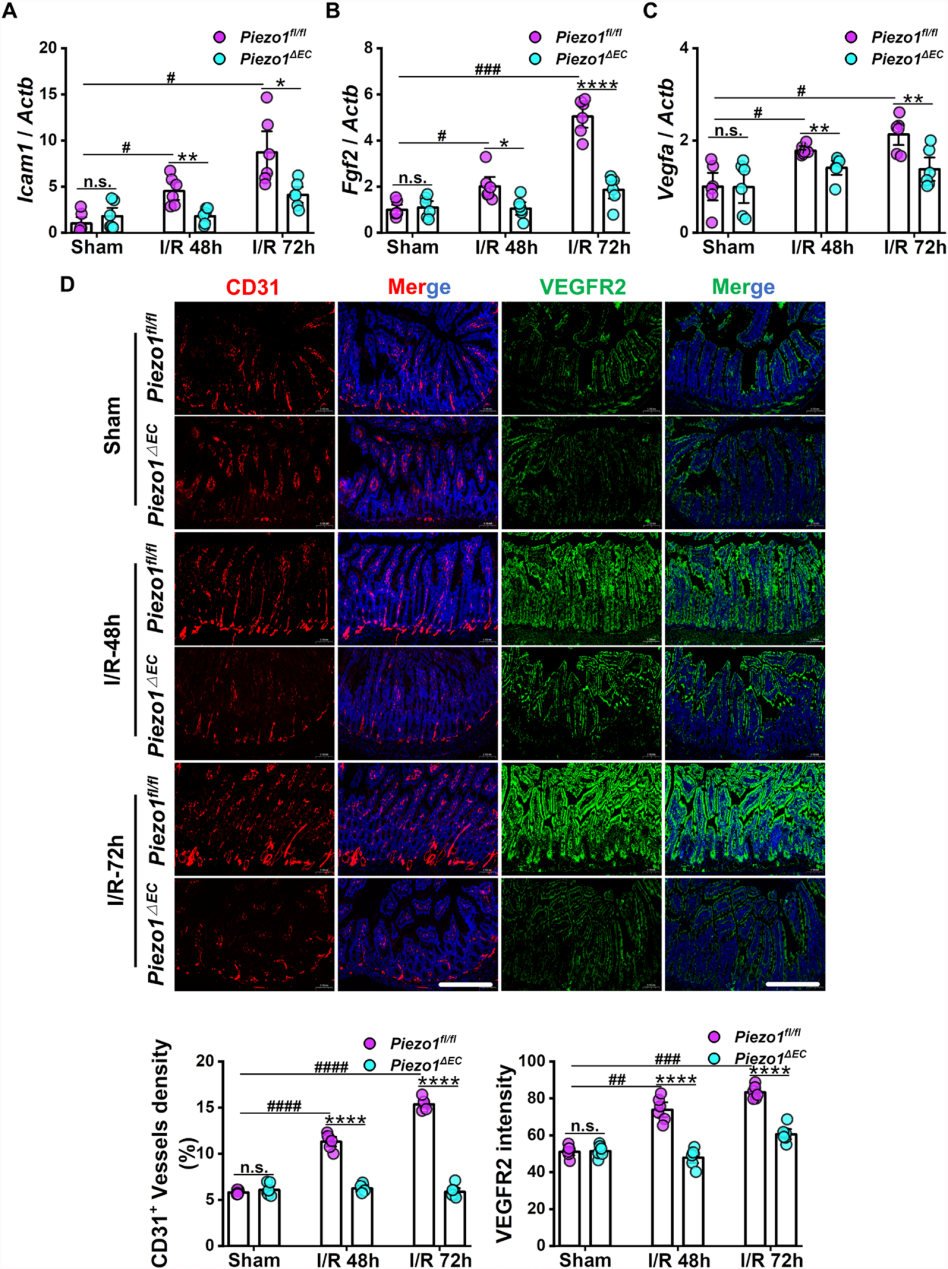
Knockout of endothelial *Piezo1* attenuates angiogenesis following intestinal I/R injury. **a–c** RT-qPCR analysis of *Icam1*, *Fgf2*, and* Vegfa* in the ileal tissues of 48 and 72 h post-I/R mice (n = 6). **d** IF staining and quantification for CD31 (red) and VEGFR2(green) in ileal sections (n = 6). Scale bar, 100 µm. Data are presented as mean ± SEM; *p < 0.05, **p < 0.01, ***p < 0.001, ****p < 0.0001, compared with *Piezo1*^*fl/fl*^ group at the same time point; ^#^*p* < 0.05, ^##^*p* < 0.01, ^###^*p* < 0.001, ^####^*p* < 0.0001, compared with Sham *Piezo1*^*fl/fl*^ group

Previous research has suggested that Piezo1 plays a role in modulating angiogenic processes (Li et al. 2014). Angiogenesis post-I/R is instrumental in facilitating the restoration of blood flow to affected areas and enhancing tissue regeneration. To further elucidate the role of Piezo1 in angiogenesis associated with intestinal I/R damage, the expression of angiogenesis associated genes in the ileal tissues were evaluated. RT-qPCR results showed that the mRNA levels of *Fgf2*, *Vegfa* and *Icam1* were increased in ileal tissues of *Piezo1*^*fl/fl*^ mice at 48 and 72 h post-I/R compared with the sham group. Compared with the *Piezo1*^*fl/fl*^ group, *Fgf2*, *Vegfa* and *Icam1* mRNA levels in the ileal tissues of *Piezo1*^*ΔEC*^ mice were decreased at the corresponding time points (Fig. 3a–c). In addition, IF staining indicated that CD31 and VEGFR2 expression levels were significantly elevated in the ileal tissues of *Piezo1*^*fl/fl*^ mice at 48 and 72 h post-I/R. However, the expression levels of CD31 and VEGFR2 were significantly decreased in the ileal tissues of *Piezo1*^*∆EC*^ mice post-I/R, compared to the *Piezo1*^*fl/fl*^ group (Fig. 3d). These results indicated that *Piezo1* knockout in endothelium results in a substantial decrease in angiogenesis following intestinal I/R injury.

### Inhibition of Piezo1 impairs H/R-induced angiogenesis in HUVECs

**Fig. 4:**
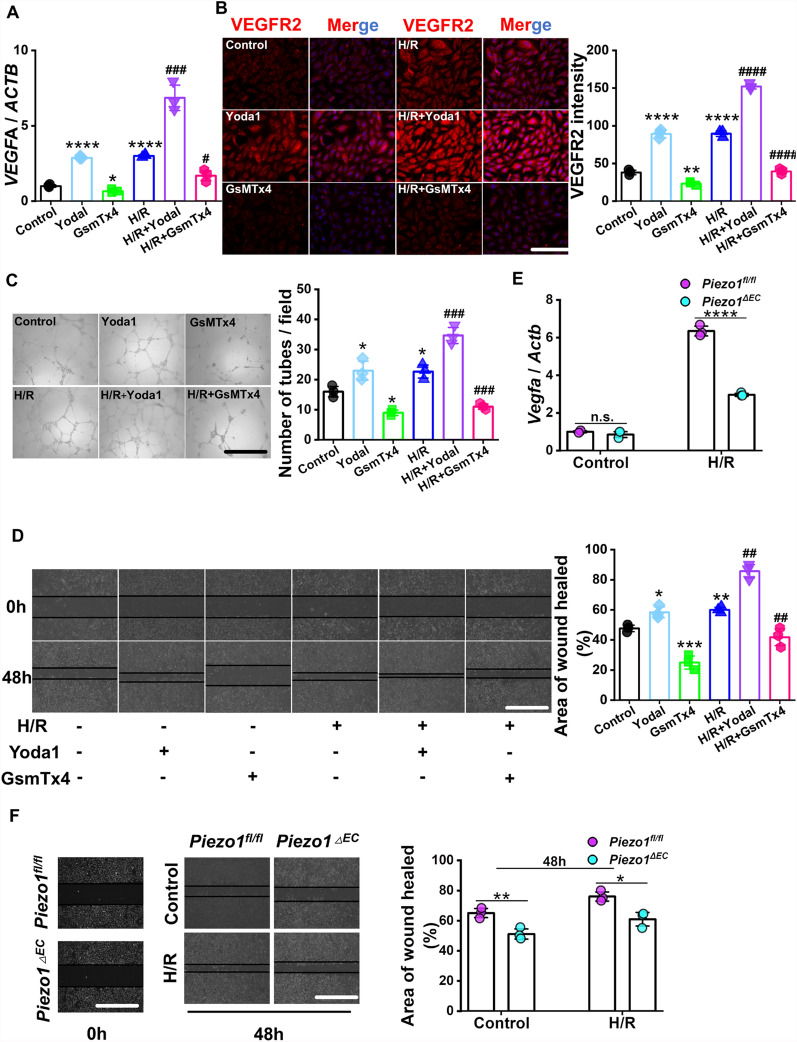
Inhibition of Piezo1 impairs H/R-induced angiogenesis in HUVECs in vitro. **a** RT-qPCR analysis of *VEGFA* expression in HUVECs (n = 3). **b** IF staining (left) and quantification (right) for VEGFR2 (red) in HUVECs (n = 3). Scale bar, 100 µm. **c** Representative images (left) and quantification (right) of tube formation assay in HEVECs (n = 3). Scale bar, 100 µm. **d** Representative images (left) and quantification (right) of scratch assay in HEVECs (n = 3). Scale bar, 100 µm. **e** RT-qPCR analysis of *Vegfa* expression in primary intestinal endothelial cells (n = 3). **f** Representative images (left) and quantification (right) of scratch assay in primary intestinal endothelial cells (n = 3). Scale bar, 100 µm. Data are presented as mean ± SEM; *p < 0.05, **p < 0.01, ***p < 0.001, ****p < 0.0001, compared with the Control group; ^#^*p* < 0.05, ^##^*p* < 0.01, ^###^*p* < 0.001, ^####^*p* < 0.0001, compared with the H/R group

Next, we examined the impact of endothelial Piezo1 on angiogenesis in a controlled environment. HUVECs were treated with H/R conditions to simulate the pathological conditions experienced by the endothelium under intestinal I/R injury. HUVECs were pretreated with 5 µM Yoda1 or 10 µM GsMTx4 for 12 h, followed by 48 h of hypoxia and 2 h of reoxygenation. The RT-qPCR analysis of *VEGFA* mRNA and IF staining of VEGFR2 protein demonstrated that activation of Piezo1 by Yoda1 resulted in an increase in their expression levels in HUVECs under normal conditions, whereas GsMTx4 treatment resulted in suppressed expression of these markers (Fig. 4a, b). Interestingly, under H/R conditions, the impact of endothelial angiogenesis was amplified by activation of Piezo1, but inhibition of Piezo1 reversed this effect (Fig. 4a, b). Consistently, the tube formation assay revealed a corresponding pattern in the total length, branch length, and number of nodes of tubes, while the scratch assay demonstrated comparable levels of cell migration (Fig. 4c, d).

We isolated primary intestinal endothelial cells from *Piezo1*^*ΔEC*^ and *Piezo1*^*fl/fl*^ mice for further validation. RT-qPCR analysis of *Vegfa* mRNA and IF staining of VEGFR2 protein revealed that knockout of Piezo1 in primary intestinal endothelial cells attenuated angiogenesis under H/R condition (Fig. 4e; Additional file 1: Fig. S2 A). Scratch assay results also indicated that knockout of *Piezo1* inhibited cell migration (Fig. 4f). In summary, our data demonstrated that endothelial *Piezo1* promotes intestinal tissue repair by regulating angiogenesis after I/R injury.

### Activation of Piezo1 induces intracellular calcium influx to promote HIF-1α-mediated angiogenesis following intestinal I/R injury

**Fig. 5:**
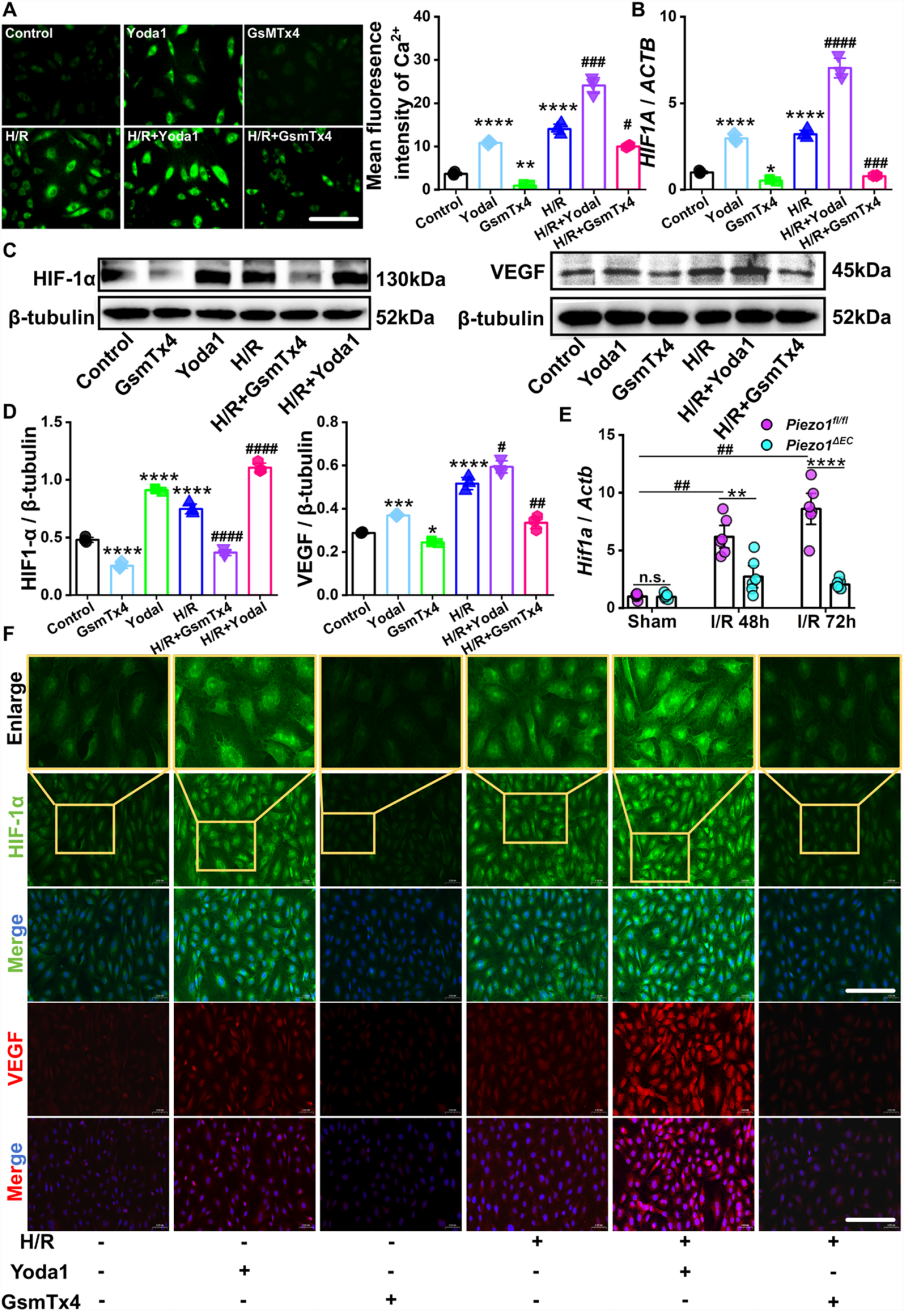
Activation of Piezo1 induces intracellular Ca^2+^ influx to promote HIF-1α-mediated angiogenesis following intestinal I/R injury. **a** Representative images (left) and quantification (right) of Fluo-4 staining in HUVECs (n = 3). Scale bar, 100 µm. **b** RT-qPCR analysis of *HIF1 A* expression in HUVECs (n = 3). **c, d** Representative WB images (upper panels) and quantifications (lower panels) of HIF-1α and VEGF in HUVECs (n = 3). **e** RT-qPCR analysis of *Hif1a* expression in ileal tissues of mice (n = 6). **f** IF staining of HIF-1α (green) and VEGF (red) in HUVECs. Scale bar, 100 µm. Data are presented as mean ± SEM; For cell samples, *p < 0.05, **p < 0.01, ***p < 0.001, ****p < 0.0001 compared with the control group; ^#^*p* < 0.05, ^##^*p*<0.01, ^###^*p* < 0.001, ^####^*p* < 0.0001 compared with the H/R group. For animal samples, *p < 0.05, **p < 0.01, ***p < 0.001, ****p < 0.0001, compared with *Piezo1*^*fl/fl*^ group at the same time point; ^#^*p* < 0.05, ^##^*p* < 0.01, ^###^*p* < 0.001, ^####^*p* < 0.0001, compared with Sham *Piezo1*^*fl/fl*^ group

In the experiments mentioned, we showed that endothelial *Piezo1* has the ability to affect intestinal I/R injury by regulating angiogenesis. Previous studies have proposed that the activation of HIF-1α is a critical step in angiogenesis under hypoxic condition (Zhang et al. 2023). Moreover, it has been established that the stabilization of HIF-1α is regulated by the Piezo1/EDN1 axis (Solis et al. 2019). Stimulation of Piezo1 results in the influx of Ca^2+^ into the cell, which triggers cellular excitation and subsequent signal transduction. We hypothesized that Ca^2+^ plays a pivotal role in effects of Piezo1-regulated angiogenesis following intestinal I/R injury. As expected, the IF analysis of intracellular concentration of Ca^2+^ demonstrated that activation of Piezo1 by Yoda1 led to an elevation in Ca^2+^ levels in HUVECs under normal conditions, whereas the introduction of GsMTx4 treatment resulted in a decreased concentration of Ca^2+^ (Fig. 5a, b). Similarly, the levels of Ca^2+^ were amplified by activation of Piezo1, but inhibition of Piezo1 reversed this effect under H/R condition (Fig. 5a, b).

To verify that Piezo1 activation mediates angiogenesis after intestinal I/R injury via HIF-1α, we measured HIF-1α expression in cells pre-stimulated with Yoda1 or GsMTx4 for 12 h, followed up by treatment with or without H/R condition for 48 h. RT-qPCR analysis of *HIF1 A* was markedly increased in Yoda1 group, but notably inhibited in GsMTx4 group, when compared to unstimulated HEVECs. Same trend was observed under H/R condition (Fig. 5c). Moreover, the mRNA expression of *Hif1a* in the ileal tissues of 48 and 72 h post-I/R *Piezo1*^*fl/fl*^ mice was markedly increased compared to *Piezo1*^*∆EC*^ group (Fig. 5d). In addition, the IF staining result indicated that, following H/R condition, there was an elevation in HIF-1α protein. Notably, the introduction of Yoda1 markedly further increased the expression of HIF-1α. Conversely, the application of GsMTx4 had an inhibitory effect on the hypoxia-induced upregulation of HIF-1α (Fig. 5e). Furthermore, the VEGF protein expression level was in agreement with the data presented in Fig. 4a (Fig. 5e). The aforementioned experimental findings have demonstrated that Piezo1, through its ability to induce Ca^2+^ influx into cells and stimulate HIF-1α expression, subsequently plays a mediatory role in angiogenesis in response to intestinal I/R injury.

### Intraperitoneal injection of Yoda1 in mice promotes angiogenesis after intestinal I/R injury

**Fig. 6:**
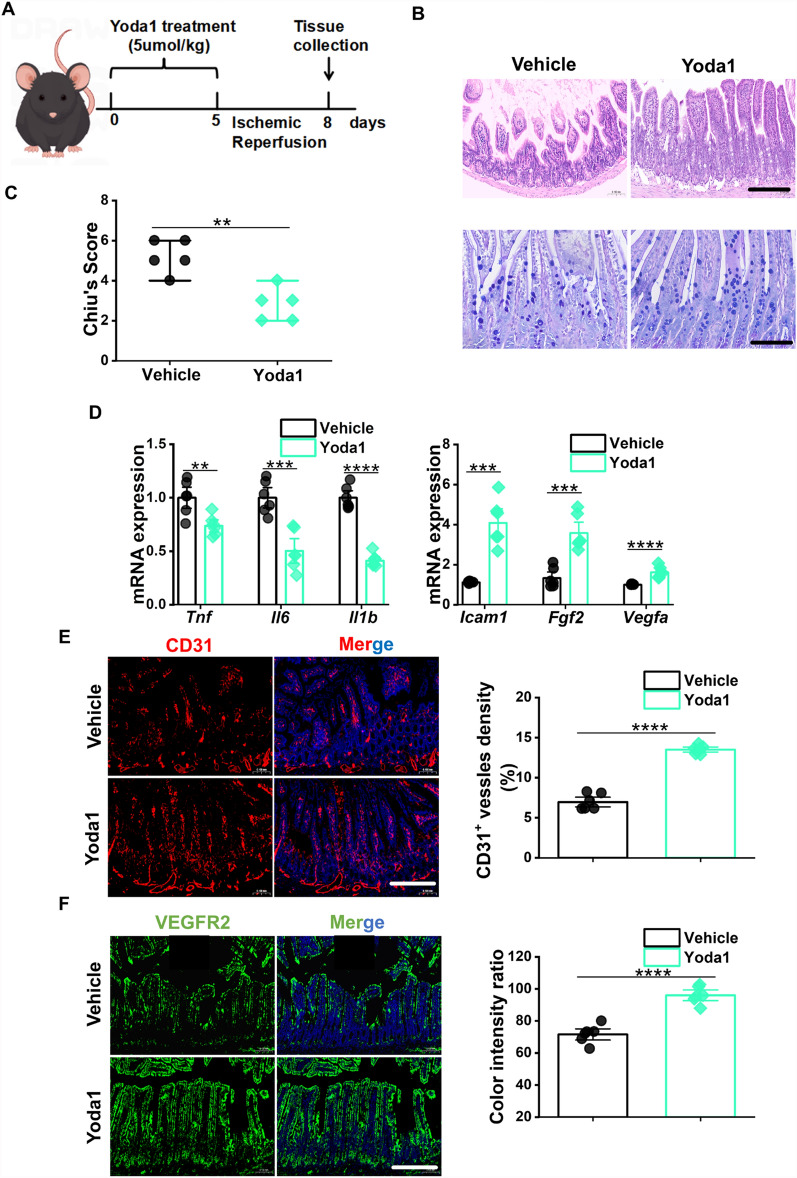
Intraperitoneal injection of Yoda1 in mice promotes angiogenesis after intestinal I/R injury. **a** Schematic illustration of the Yoda1 treatment in post-intestinal I/R C57BL/6 J mice. **b** Representative images of H&E and AB-PAS staining in ileal slices of C57BL/6 J mice. Scale bar, 100 µm. **c** Histopathological scoring (Chiu's score) of ileal tissues as shown in (**a**) (n = 6). **d** RT-qPCR analysis of *Tnf*, *Il6*, *Il1b, Icam1*, *Fgf2*, and *Vegfa* in the ileal tissues of C57BL/6 J mice (n = 6). **e** IF staining (left) and quantification (right) for CD31 (red) in ileal sections of C57BL/6 J mice (n = 6). Scale bar, 100 µm. **f** IF staining (left) and quantification (right) for VEGFR2 (green) in ileal sections of C57BL/6 J mice (n = 6). Scale bar, 100 µm. Data are presented as mean ± SEM; **p < 0.01, ***p < 0.001, ****p < 0.0001 compared with the Vehicle group

**Fig. 7:**
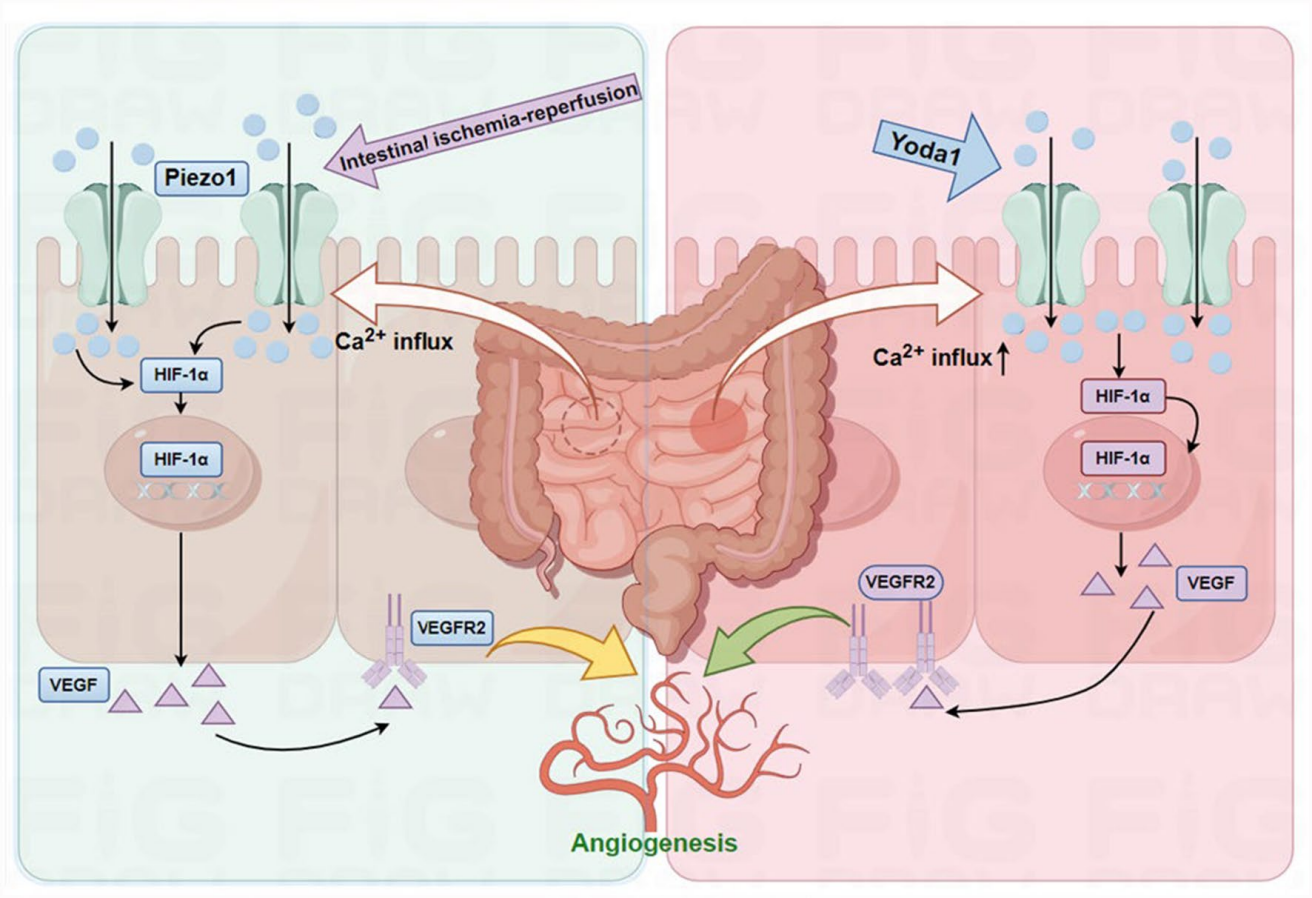
Schematic illustration of the mechanosensitive ion channel Piezo1 in the regulation of angiogenesis following intestinal post-I/R injury

Yoda1, a small molecule compound, selectively activates Piezo1. To provide a clearer understanding of the function of endothelial Piezo1 in the process of angiogenesis during intestinal I/R injury, Yoda1 was administered intraperitoneally to C57BL/6 J mice, and ileal tissues were collected after 72 h post-I/R for further experiments (Fig. 6a). Interestingly, H&E and AB-PAS staining revealed that Yoda1-treated post-I/R mice exhibited mild damage to intestinal villi and higher concentration of mucin in the ileum than I/R group (Fig. 6b). The Chiu's scores of ileal slices in the Yoda1-treated post-I/R mice were lower than those in the I/R group (Fig. 6c). Furthermore, the post-I/R + Yoda1 group exhibited notably increased expression of *Icam1*,* Vegfa*, and *Fgf2* compared to the post-I/R group. However, in comparison to the post-I/R + Yoda1 group, the post-I/R mice exhibited a marked upregulation of these inflammatory genes (Fig. 6d). Consistent with our expectations, the immunofluorescence staining for CD31 and VEGFR2 revealed that the post-I/R + Yoda1 group had a notably greater degree of angiogenesis in their ileal tissues than the post-I/R mice (Fig. 6e, f). Our data collectively suggested that endothelial Piezo1 is involved in modulating angiogenesis after intestinal I/R injury, and that the activation of Piezo1 by Yoda1 amplifies the angiogenic response in the ileum post-I/R injury (Fig. 7).

## Discussion

The objective of the current study was to clarify the function and fundamental mechanisms of Piezo1 in intestinal I/R injury. Our experimental findings demonstrated that there was a significant increasing expression of intestinal endothelial Piezo1 post I/R injury, which suggested that Piezo1 is crucial in the response to intestinal I/R injury. Upregulation of Piezo1 may represent a cellular adaptive response to mechanical stress, conducive to vascular repair and regeneration. In the absence of *Piezo1* post-I/R injury, experiments with endothelial cell-specific *Piezo1* knockout mice revealed exacerbated intestinal injury, villous denudation, reduced mucin secretion, and diminished angiogenesis. Intraperitoneal administration of the Piezo1 agonist Yoda1 in mice reduced the levels of inflammatory response, ameliorated I/R-induced intestinal injury, and concurrently promoted the upregulation of *Vegfa*, *Fgf2*, and *Icam1* gene expressions, facilitating angiogenesis following I/R injury. In vitro experiments also confirmed that inhibition of Piezo1 impacted H/R-induced angiogenesis in HUVECs. Activation of the Piezo1 ion channel led to increased Ca^2+^ influx, triggering downstream transcription factor HIF-1α activation, which in turn induced upregulation of VEGF expression, promoting neovascularization.

Angiogenesis, orchestrated by endothelial cells, constitutes an intricate process following I/R injury. In a hypoxic or inflammatory microenvironment, vascular endothelial cells have the capacity to secrete VEGF, which is an important pro-angiogenic mediator and triggers the degradation of the basement membrane, thereby facilitating the formation of novel endothelial sprouts (Sakai et al. 2023). Existing research underscores the beneficial effects of endothelial angiogenesis on the repair of ischemic tissues in the heart, brain, kidney, and liver (Zhang et al. 2024; Li et al. 2024; Ogino et al. 2024; Wang et al. 2023). However, the function of the vascular endothelium in the context of intestinal I/R injury has received relatively limited research attention. Our study showed a notable elevation in the count of CD31^+^ cells and VEGF expression levels at 48 and 72 h following intestinal I/R injury, indicating that angiogenesis is crucial in the pathophysiological processes of intestinal I/R injury.

Piezo1, a mechanosensitive ion channel, exhibits differential expression across various intestinal cell types, such as intestinal epithelial cells and intestinal L cells, contributing to a multitude of physiological functions (He et al. 2024). Intestinal epithelial cells express Piezo1, which participates in digestion, absorption, and secretion processes. Furthermore, the expression of Piezo1 within intestinal epithelial cells may be important in sustaining the epithelial barrier function and maintaining dynamic equilibrium, both of which are essential for protecting against external environmental stressors and preventing the onset of tumorigenesis (He et al. 2023). Furthermore, the presence of Piezo1 in intestinal L cells is intimately linked to maintaining energy balance and regulating glucose levels. Mice lacking Piezo1 specifically in L cells show decreased glucose tolerance and lowered GLP-1 production, both under standard and high-fat dietary conditions, hinting that Piezo1 may regulate glucose levels by adjusting the production and release of GLP-1 (Huang et al. 2024). On the contrary, our IF staining data showed that expression of Piezo1 is mainly upregulated in endothelial cells, not in epithelial cells or intestinal L cells during intestinal I/R injury, which indicates the important role of endothelial Piezo1 during intestinal I/R injury. Indeed, further experiments are needed to explore the expression and the role of epithelial or L cell Piezo1 in I/R injury.

During I/R injury, the activation of endothelial Piezo1 potentially implicates a variety of pathways. During ischemia–reperfusion, vascular endothelial cells can sense various abnormal signals, including mechanical signals such as changes in osmotic pressure and hemodynamic abnormalities, as well as chemical signals such as changes in inflammatory factors, ATP levels, and pH values (Yue et al. 2024; Lim et al. 2024; Martins et al. 2016). These signals are potential pathways to activate the Piezo1 channel. Several studies have confirmed the relationship between Piezo1 and angiogenesis (Chen et al. 2021; Liu et al. 2024). Consistent with previous research, endothelial Piezo1 is responsible for angiogenesis because Piezo1 knockout in vascular endothelial cells diminished the process, leading to accelerated intestinal I/R injury.

Increased substrate stiffness leads to the upregulation of Piezo1, which subsequently prevents the ubiquitination of HIF-1α, then boosts the expression of downstream pro-angiogenic factors, thereby accelerating the angiogenesis process (Li et al. 2022). HIF-1α is a pivotal transcription factor. When oxygen levels are low, the rate of HIF-1α degradation decreases, enabling it to accumulate in the nucleus and unite with the HIF-1β subunit to create the HIF-1 complex (Weidemann and Johnson 2008). The HIF-1 complex has the ability to attach to hypoxia response elements (HREs), which triggers the expression of downstream target genes, such as VEGF. The activation of HIF-1α serves as a pivotal upstream event in the synthesis of VEGF-A and constitutes a crucial component of the hypoxia-sensing pathway (Ahn et al. 2014). It promotes rapid angiogenesis and vascular formation in hypoxic tissues, not only aiding in the generation of new blood vessels but also participating in vascular remodeling and maturation processes (Ahluwalia and Tarnawski 2012). Among the genes regulated by HIF-1α, there are also other factors that are associated with angiogenesis, including transforming growth factor-beta (TGF-β) and platelet-derived growth factor (PDGF) (Lei et al. 2024; Sun et al. 2020). Our cellular experiments have confirmed that, under hypoxic conditions, the activation of Piezo1 ion channels results in an influx of Ca^2^⁺, which in turn activates HIF-1α, subsequently promoting the production of VEGF. However, owing to temporal limitations, the underlying mechanisms by which HIF-1α further enhances the expression of angiogenic factors, like VEGF, are still not fully understood.

## Conclusion

In conclusion, our rigorous investigation has conclusively demonstrated that the activation of Piezo1 ion channels elicits a significant intracellular influx of Ca^2^⁺, which subsequently stimulates angiogenesis through the mediation of HIF-1α. This pivotal discovery provides deep insights into the intricate molecular mechanisms underlying Piezo1's role in angiogenesis, thereby offering promising avenues for future investigative endeavors. The sequential events of Ca^2^⁺ influx and HIF-1α activation represent a crucial signaling axis through which Piezo1 exerts its angiogenic enhancing effects. Our results are in consonance with the contemporary scientific consensus regarding the functions of Piezo1 in angiogenesis and tissue injury, thereby adding to the robustness of the existing knowledge base. Most importantly, our study marks a significant advancement as it is the inaugural evaluation of the protective roles of Piezo1 in intestinal I/R injury and by experimentally substantiating the therapeutic potential of Piezo1 activators, thus paving the way for innovative therapeutic strategies.

## Supplementary Information

Additional file 1. Figure S1. Validation of endothelial *Piezo1* knockout mice.Gel electrophoresis of PCR endpoint products. The top panel demonstrated the presence of the Cre recombinase transgene, while the bottom panel confirmed the presence of LoxP sequenceswithin the Piezo1 gene.RTq-PCR analysis of *Piezo1* abundance in murine intestinal endothelial cells from *Piezo1*^*fl/fl*^ and *Piezo1*^*ΔEC*^ mice.Representative WB images of Piezo1 in murine intestinal endothelial cells from *Piezo1*^*fl/fl*^ and *Piezo1*^*ΔEC*^ mice.Representative traces of intracellular Ca^2+^ changes (△) in murine intestinal endothelial cells from *Piezo1*^*fl/fl*^ and *Piezo1*^*ΔEC*^ mice in response to 5 µM Yoda1.Peak value of Ca^2+^ in murine intestinal endothelial cells from *Piezo1*^*fl/fl*^ and *Piezo1*^*ΔEC*^ mice in response to 5 μM Yoda1. Data are presented as mean ± SEM; ****P < 0.0001, compared with *Piezo1*^*fl/fl*^ group. Figure S2. Validation of the angiogenic effect of Piezo1 in primary intestinal endothelial cells.: Immunofluorescence staining of VEGFR2 in primary intestinal endothelial cells. Scale bar, 100 µm.

## Data Availability

No datasets were generated or analysed during the current study.

## Abbreviations

AB-PAS: Alcian blue-periodic acid-Schiff
FGF: Fibroblast growth factor
H&E: Hematoxylin and eosin
HIF: Hypoxia-inducible factor
H/R: Hypoxia/reoxygenation
HUVECs: Human umbilical vein endothelial cells
IF: Immunofluorescence
ICAM: Intercellular cell adhesion molecule
IL: Interleukin
I/R: Ischemia–reperfusion
PDGF: Platelet-derived growth factor
TGF: Transforming growth factor
TNF: Tumor necrosis factor
VEGF: Vascular endothelial growth factor

## References

[CR1] Ahluwalia A, Tarnawski AS: Critical role of hypoxia sensor--HIF-1α in VEGF gene activation. Implications for angiogenesis and tissue injury healing. Curr Med Chem. 2012;19(1):90–7. 10.2174/092986712803413944 22300081

[CR2] Ahn GO, Seita J, Hong BJ, Kim YE, Bok S, Lee CJ, Kim KS, Lee JC, Leeper NJ, Cooke JP, et al. Transcriptional activation of hypoxia-inducible factor-1 (HIF-1) in myeloid cells promotes angiogenesis through VEGF and S100A8. Proc Natl Acad Sci U S A. 2014;111(7):2698–703. 24497508 10.1073/pnas.1320243111 PMC3932909

[CR3] Bentley K, Chakravartula S. The temporal basis of angiogenesis. Philos Trans R Soc Lond B Biol Sci. 2017;372(1720):20150522. 10.1098/rstb.2015.0522 PMC5379027 28348255

[CR4] Chen P, Zhang G, Jiang S, Ning Y, Deng B, Pan X, Liu S, He Y, Zhang L, Wan R, et al. Mechanosensitive Piezo1 in endothelial cells promotes angiogenesis to support bone fracture repair. Cell Calcium. 2021;97: 102431. 34153657 10.1016/j.ceca.2021.102431

[CR5] Chiba T, Cerqueira DM, Li Y, Bodnar AJ, Mukherjee E, Pfister K, Phua YL, Shaikh K, Sanders BT, Hemker SL, et al. Endothelial-derived miR-17∼92 promotes angiogenesis to protect against renal ischemia-reperfusion injury. J Am Soc Nephrol. 2021;32(3):553–62. 33514560 10.1681/ASN.2020050717 PMC7920169

[CR6] Deng F, Lin ZB, Sun QS, Min Y, Zhang Y, Chen Y, Chen WT, Hu JJ, Liu KX. The role of intestinal microbiota and its metabolites in intestinal and extraintestinal organ injury induced by intestinal ischemia reperfusion injury. Int J Biol Sci. 2022;18(10):3981–92. 35844797 10.7150/ijbs.71491 PMC9274501

[CR7] Dudley AC, Griffioen AW. The modes of angiogenesis: an updated perspective. Angiogenesis. 2023;26(4):477–80. 37640982 10.1007/s10456-023-09895-4 PMC10777330

[CR8] Gubernatorova EO, Perez-Chanona E, Koroleva EP, Jobin C, Tumanov AV. Murine Model of Intestinal Ischemia-reperfusion Injury. J Vis Exp. 2016;2016(111). 10.3791/53881 PMC4942093 27213580

[CR9] He H, Zhou J, Xu X, Zhou P, Zhong H, Liu M. Piezo channels in the intestinal tract. Front Physiol. 2024;15:1356317. 38379701 10.3389/fphys.2024.1356317 PMC10877011

[CR10] He J, Xie X, Xiao Z, Qian W, Zhang L, Hou X. Piezo1 in digestive system function and dysfunction. Int J Mol Sci. 2023;24(16):12953. 10.3390/ijms241612953 PMC10454946 37629134

[CR11] Huang Y, Mo H, Yang J, Gao L, Tao T, Shu Q, Guo W, Zhao Y, Lyu J, Wang Q et al. Mechano-regulation of GLP-1 production by Piezo1 in intestinal L cells. Elife. 2024. 10.7554/eLife.97854.3 10.7554/eLife.97854 PMC11542922 39509292

[CR12] Kang H, Hong Z, Zhong M, Klomp J, Bayless KJ, Mehta D, Karginov AV, Hu G, Malik AB. Piezo1 mediates angiogenesis through activation of MT1-MMP signaling. Am J Physiol Cell Physiol. 2019;316(1):C92-c103. 30427721 10.1152/ajpcell.00346.2018 PMC6383143

[CR13] La Mendola D, Trincavelli ML, Martini C. Angiogenesis in disease. Int J Mol Sci. 2022;23(18):10962. 10.3390/ijms231810962 PMC9503835 36142885

[CR14] Lai A, Cox CD, Chandra Sekar N, Thurgood P, Jaworowski A, Peter K, Baratchi S. Mechanosensing by Piezo1 and its implications for physiology and various pathologies. Biol Rev Camb Philos Soc. 2022;97(2):604–14. 34781417 10.1111/brv.12814

[CR15] Lei J, Jiang X, Huang D, Jing Y, Yang S, Geng L, Yan Y, Zheng F, Cheng F, Zhang W, et al. Human ESC-derived vascular cells promote vascular regeneration in a HIF-1α dependent manner. Protein Cell. 2024;15(1):36–51. 37158785 10.1093/procel/pwad027 PMC10762672

[CR16] Li J, Hou B, Tumova S, Muraki K, Bruns A, Ludlow MJ, Sedo A, Hyman AJ, McKeown L, Young RS, et al. Piezo1 integration of vascular architecture with physiological force. Nature. 2014;515(7526):279–82. 25119035 10.1038/nature13701 PMC4230887

[CR17] Li S, Zhou Y, Gu X, Zhang X, Jia Z. NLRX1/FUNDC1/NIPSNAP1-2 axis regulates mitophagy and alleviates intestinal ischaemia/reperfusion injury. Cell Prolif. 2021;54(3): e12986. 33432610 10.1111/cpr.12986 PMC7941235

[CR18] Li M, Zhang X, Wang M, Wang Y, Qian J, Xing X, Wang Z, You Y, Guo K, Chen J, et al. Activation of Piezo1 contributes to matrix stiffness-induced angiogenesis in hepatocellular carcinoma. Cancer Commun (Lond). 2022;42(11):1162–84. 36181398 10.1002/cac2.12364 PMC9648387

[CR19] Li PB, Bai JQ, Jiang WX, Li HH, Li CM. The mechanosensitive Piezo1 channel exacerbates myocardial ischaemia/reperfusion injury by activating caspase-8-mediated PANoptosis. Int Immunopharmacol. 2024;139: 112664. 39008937 10.1016/j.intimp.2024.112664

[CR20] Lim XR, Abd-Alhaseeb MM, Ippolito M, Koide M, Senatore AJ, Plante C, Hariharan A, Weir N, Longden TA, Laprade KA, et al. Endothelial Piezo1 channel mediates mechano-feedback control of brain blood flow. Nat Commun. 2024;15(1):8686. 39375369 10.1038/s41467-024-52969-0 PMC11458797

[CR21] Liu H, Zhou H, Fan Y, Li J, Guo Z, Xu Q, Liu Y, Gao K, Lahcine NA, Zhang J, et al. Macrophages regulate angiogenesis-osteogenesis coupling induced by mechanical loading through the Piezo1 pathway. J Bone Miner Res. 2024. 10.1093/jbmr/zjae198. 10.1093/jbmr/zjae198 39657223

[CR22] Luo S, Zhao X, Jiang J, Deng B, Liu S, Xu H, Tan Q, Chen Y, Zhang Z, Pan X, et al. Piezo1 specific deletion in macrophage protects the progression of liver fibrosis in mice. Theranostics. 2023;13(15):5418–34. 37908726 10.7150/thno.86103 PMC10614683

[CR23] Ma S, Dubin AE, Zhang Y, Mousavi SAR, Wang Y, Coombs AM, Loud M, Andolfo I, Patapoutian A. A role of PIEZO1 in iron metabolism in mice and humans. Cell. 2021;184(4):969-982.e913. 33571427 10.1016/j.cell.2021.01.024 PMC7927959

[CR24] Martins JR, Penton D, Peyronnet R, Arhatte M, Moro C, Picard N, Kurt B, Patel A, Honoré E, Demolombe S. Piezo1-dependent regulation of urinary osmolarity. Pflugers Arch. 2016;468(7):1197–206. 27023350 10.1007/s00424-016-1811-z

[CR25] Ogino S, Yoshikawa K, Nagase T, Mikami K, Nagase M. Roles of the mechanosensitive ion channel Piezo1 in the renal podocyte injury of experimental hypertensive nephropathy. Hypertens Res. 2024;47(3):747–59. 38145990 10.1038/s41440-023-01536-z

[CR26] Sakai K, Hayashi T, Sakai Y, Mada J, Tonami K, Uchijima Y, Kurihara H, Tokihiro T. A three-dimensional model with two-body interactions for endothelial cells in angiogenesis. Sci Rep. 2023;13(1):20549. 37996513 10.1038/s41598-023-47911-1 PMC10667370

[CR27] Solis AG, Bielecki P, Steach HR, Sharma L, Harman CCD, Yun S, de Zoete MR, Warnock JN, To SDF, York AG, et al. Mechanosensation of cyclical force by PIEZO1 is essential for innate immunity. Nature. 2019;573(7772):69–74. 31435009 10.1038/s41586-019-1485-8 PMC6939392

[CR28] Sun Y, Li M, Liu G, Zhang X, Zhi L, Zhao J, Wang G. The function of Piezo1 in colon cancer metastasis and its potential regulatory mechanism. J Cancer Res Clin Oncol. 2020;146(5):1139–52. 32152662 10.1007/s00432-020-03179-w PMC7142063

[CR29] Wang YY, Zhang H, Ma T, Lu Y, Xie HY, Wang W, Ma YH, Li GH, Li YW. Piezo1 mediates neuron oxygen-glucose deprivation/reoxygenation injury via Ca(2+)/calpain signaling. Biochem Biophys Res Commun. 2019;513(1):147–53. 30948157 10.1016/j.bbrc.2019.03.163

[CR30] Wang Q, Peng X, Chen Y, Tang X, Qin Y, He M, Chen W, Chen H. Piezo1 alleviates acetaminophen-induced acute liver injury by activating Nrf2 and reducing mitochondrial reactive oxygen species. Biochem Biophys Res Commun. 2023;652:88–94. 36841099 10.1016/j.bbrc.2023.02.043

[CR31] Watson MJ, Ke B, Shen XD, Gao F, Busuttil RW, Kupiec-Weglinski JW, Farmer DG. Intestinal ischemia/reperfusion injury triggers activation of innate toll-like receptor 4 and adaptive chemokine programs. Transplant Proc. 2008;40(10):3339–41. 19100385 10.1016/j.transproceed.2008.07.144 PMC2975481

[CR32] Wei J, Xie J, He J, Li D, Wei D, Li Y, Li X, Fang W, Wei G, Lai K. Active fraction of Polyrhachis vicina (Roger) alleviated cerebral ischemia/reperfusion injury by targeting SIRT3-mediated mitophagy and angiogenesis. Phytomedicine. 2023;121: 155104. 37797433 10.1016/j.phymed.2023.155104

[CR33] Weidemann A, Johnson RS. Biology of HIF-1alpha. Cell Death Differ. 2008;15(4):621–7. 18259201 10.1038/cdd.2008.12

[CR34] Yue Y, Chen P, Ren C. Piezo1 modulates neuronal autophagy and apoptosis in cerebral ischemia-reperfusion injury through the AMPK-mTOR signaling pathway. Neurochem Res. 2024;50(1):32. 39585469 10.1007/s11064-024-04291-w

[CR35] Zhang X, Hou L, Li F, Zhang W, Wu C, Xiang L, Li J, Zhou L, Wang X, Xiang Y, et al. Piezo1-mediated mechanosensation in bone marrow macrophages promotes vascular niche regeneration after irradiation injury. Theranostics. 2022;12(4):1621–38. 35198061 10.7150/thno.64963 PMC8825582

[CR36] Zhang Y, Cao Y, Li Y, Xiao L, Xu W, Xu W, Huang M, Zhang X, Chen Y, Nan L. Gualou Guizhi decoction promotes therapeutic angiogenesis via the miR210/HIF/VEGF pathway in vivo and in vitro. Pharm Biol. 2023;61(1):779–89. 37158290 10.1080/13880209.2023.2204142 PMC10171115

[CR37] Zhang M, Liu Q, Meng H, Duan H, Liu X, Wu J, Gao F, Wang S, Tan R, Yuan J. Ischemia-reperfusion injury: molecular mechanisms and therapeutic targets. Signal Transduct Target Ther. 2024;9(1):12. 38185705 10.1038/s41392-023-01688-x PMC10772178

