# Supplementary material for: Endothelial Piezo1 stimulates angiogenesis to offer protection against intestinal ischemia–reperfusion injury in mice
Source: Mol Med. 2025 Apr 22;31:147. doi: 10.1186/s10020-025-01197-3 (PMC12016420; doi:10.1186/s10020-025-01197-3)
Supplement: Supplementary file 1 — Additional file 1. Figure S1. Validation of endothelial Piezo1 knockout mice.Gel electrophoresis of PCR endpoint products. The top panel demonstrated the presence of the Cre recombinase transgene, while the bottom panel confirmed the presence of LoxP sequenceswithin the Piezo1 gene.RTq-PCR analysis of Piezo1 abundance in murine intestinal endothelial cells from Piezo1fl/fl and Piezo1ΔEC mice.Representative WB images of Piezo1 in murine intestinal endothelial cells from Piezo1fl/fl and Piezo1ΔEC mice.Representative traces of intracellular Ca2+ changes (△) in murine intestinal endothelial cells from Piezo1fl/fl and Piezo1ΔEC mice in response to 5 µM Yoda1.Peak value of Ca2+ in murine intestinal endothelial cells from Piezo1fl/fl and Piezo1ΔEC mice in response to 5 μM Yoda1. Data are presented as mean ± SEM; ****P < 0.0001, compared with Piezo1fl/fl group. Figure S2. Validation of the angiogenic effect of Piezo1 in primary intestinal endothelial cells.: Immunofluorescence staining of VEGFR2 in primary intestinal endothelial cells. Scale bar, 100 µm. [file 10020_2025_1197_MOESM1_ESM.docx]

**Supplement materials**

**Endothelial Piezo1 stimulates angiogenesis to offer protection against intestinal ischemia-reperfusion injury in mice**

**Cuifen Wang**^1, #^, **Shangfei Luo**^1, #^, **Yameng Yan**^1, #^, **Jinze Li**^1, #^, Weipin Niu^1^, Tianying Hong^1^, Kai Hao^1^, Xin Sun^1^, Jiali Liu^1^, Ran An^3^, Jing Li^1, 2, *^

^1^ Innovation Research Center, Shandong University of Chinese Medicine, Jinan, 250307, China.

^2^ The First Affiliated Hospital, Guangzhou University of Chinese Medicine, Guangzhou, 510405, China.

^3^ Pacific College of Health and Science, 110 William St 19th floor, New York, NY 10038, United State of America.

^#^ The authors contribute equally.

^*^ Corresponding author.

**Corresponding author:**

Jing Li, PhD,

Innovation Research Center, Shandong University of Chinese Medicine, Jinan, 250307, China; The First Affiliated Hospital, Guangzhou University of Chinese Medicine, Guangzhou, 510405, China.

Email: bmsjingl@gzucm.edu.cn

**
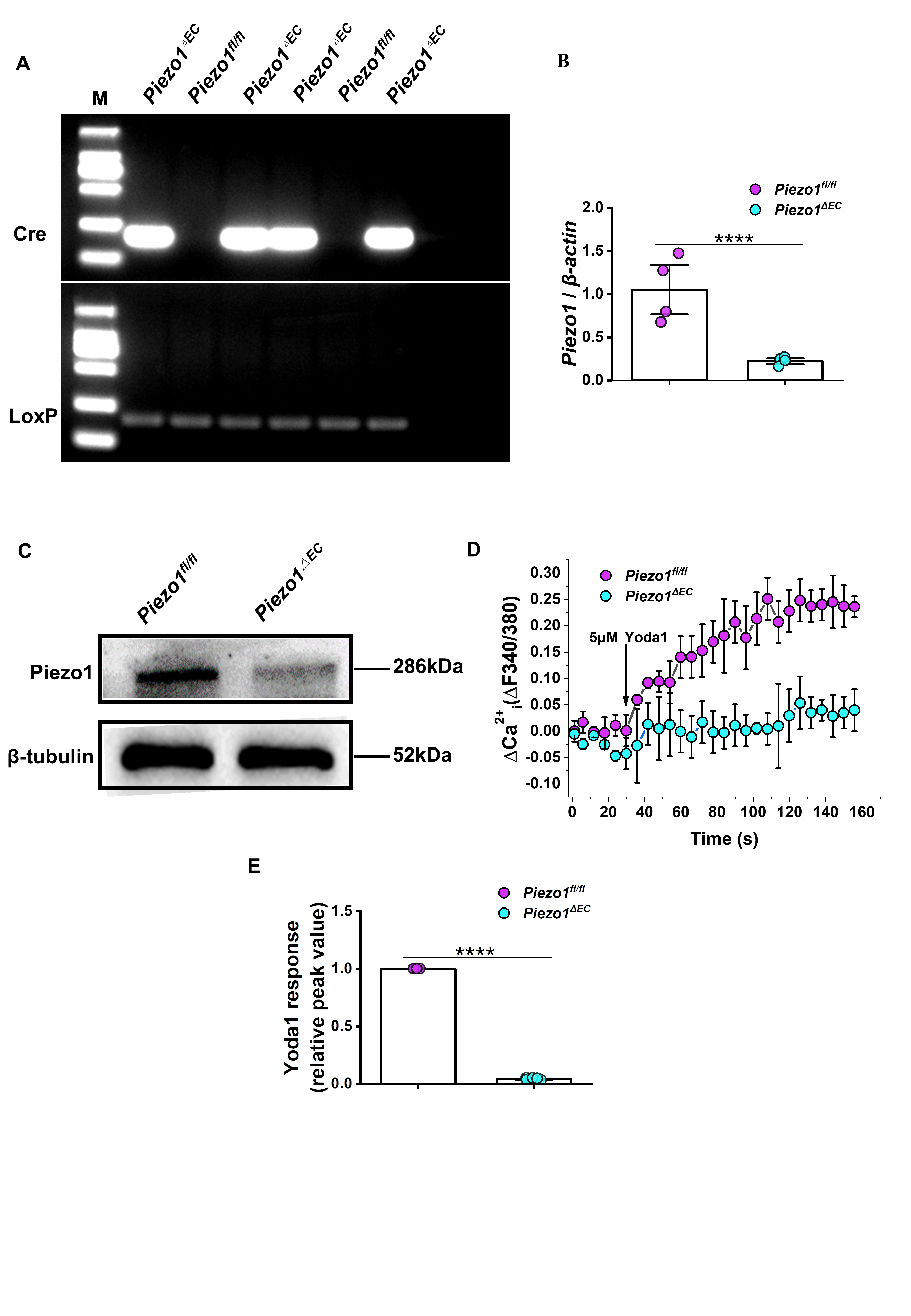
**

**Figure S1: Validation of endothelial *Piezo1* knockout mice**

(A) Gel electrophoresis of PCR endpoint products. The top panel demonstrated the presence of the Cre recombinase transgene (191bp), while the bottom panel confirmed the presence of LoxP sequences (189bp) within the Piezo1 gene.

(B) RTq-PCR analysis of *Piezo1* abundance in murine intestinal endothelial cells from *Piezo1^fl/fl^* and *Piezo1^ΔEC^* mice (n = 6).

(C) Representative WB images of Piezo1 in murine intestinal endothelial cells from *Piezo1^fl/fl^* and *Piezo1^ΔEC^* mice.

(D) Representative traces of intracellular Ca^2+^ changes (Δ) in murine intestinal endothelial cells from *Piezo1^fl/fl^* and *Piezo1^ΔEC^* mice in response to 5 µM Yoda1.

(E) Peak value of Ca^2+^ in murine intestinal endothelial cells from *Piezo1^fl/fl^* and *Piezo1^ΔEC^* mice in response to 5 μM Yoda1 (n = 6).

Data are presented as mean ± SEM; *****P < 0.0001*, compared with *Piezo1^fl/fl^* group*.*


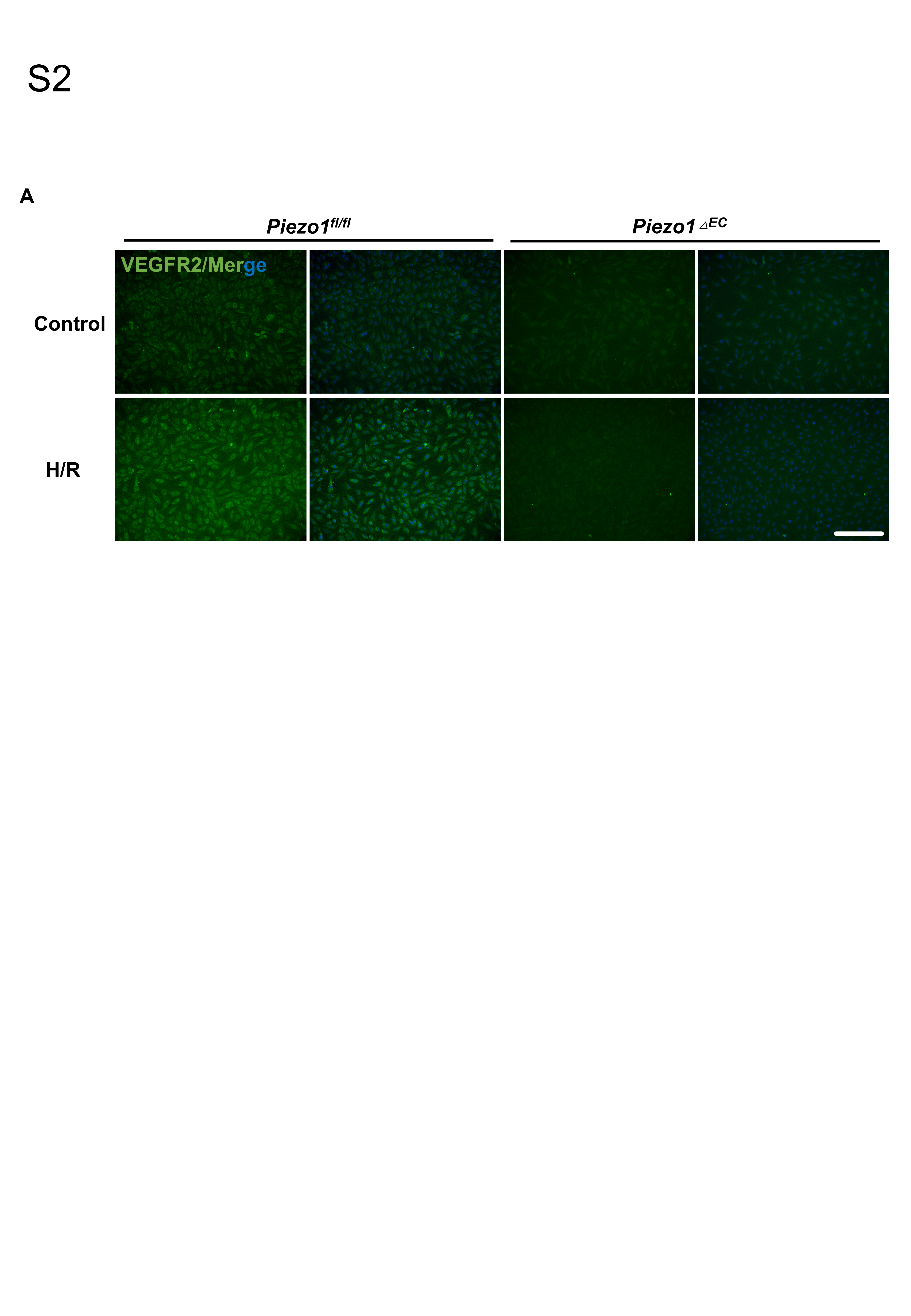


**Figure S2: Validation of the angiogenic effect of Piezo1 in primary intestinal endothelial cells.**

(A): Immunofluorescence staining of VEGFR2 (green) in primary intestinal endothelial cells. Scale bar, 100 µm.
